# Payment by results in forensic mental health

**DOI:** 10.1192/pb.bp.114.048330

**Published:** 2015-10

**Authors:** Luke Gibbons, Lucy McCarthy

**Affiliations:** 1 Nottinghamshire Healthcare NHS Trust

## Abstract

Forensic mental health services are low-volume, high-cost services. Payment by results (PbR) is the UK s latest attempt to improve efficiency and controls pending behaviours within the secure services. This article discusses the utility of the PbR mechanic in forensic mental health. It explores PbR implementation in non-forensic mental health settings, similar funding processes internationally, and early PbR implementation work in the UK's secure services. Finally, the article discusses the challenges faced when implementing PbR in forensic mental health services and puts forward possible next steps in determining the utility of PbR in forensic mental health.

The majority of mental health services in England, including forensic mental health services, have previously been funded via block contracts, with annual budgets set and agreed between commissioners and service providers. Providers then distribute the finances, balancing governmental directives and strategies against local priorities. However, it has been reported that block contracting offers very little incentive to improve efficiency and control spending behaviours.^1^ Payment by results (PbR), soon to be called pricing and currency, is ‘the payment system in England under which commissioners pay healthcare providers for each patient seen or treated, taking into account the complexity of the patient's healthcare needs’^2^ that attempts to address such issues. It is England's answer to a case-mix approach, in which funding involves the use of a predefined average care package, which is applied with a fixed price when certain diagnoses or factors are present. In 2003, this method of commissioning started to be rolled out in areas of physical healthcare and now the PbR mechanism is utilised in the bulk of elective in-patient/out-patient and emergency procedures. The government believes that it will provide more clarity on what to expect from services and achieve better, more efficient outcomes.^3^

## PbR: key concepts

Within the scheme, when operating for physical health purposes, a currency represents a unit of healthcare for which a payment is made and a tariff represents the price paid for each currency. Consequently, varying patients are grouped into healthcare resource groups (HRGs), in accordance with similar consumption of National Health Service (NHS) resources. In mental health services, ‘clusters’ are used as the so-called currencies, which in turn define a relevant care pathway and ultimately the contract between commissioners and service providers.^4^ Similar to HRGs, clusters represent individuals with similar needs, requiring similar resources. Whether the PbR mechanism can be applied to a mental health domain is questionable. Owing to the complex nature of human condition and the often long-term nature of mental disorders, it would be naive to think that categorising mental health patients into discrete groups would be as simple as categorising patients with similar physical health problems.^5^ Consequently, any success of the PbR mechanism in the physical health domain does not necessarily mean that it is transferable to a mental health domain.

### Are the positive effects of PbR smoke and mirrors? Evidence from non-psychiatric settings

Several countries operate mechanisms that attempt to match resources more directly to measured needs. This particular approach seems sensible for both commissioners and healthcare providers, as theoretically hospitals are prospectively reimbursed in accordance with diagnosis. Thus, any additional funding needed for a mismatch between diagnosis and service will result in financial loss for the hospital. Alternatively, any funds left unspent will result in profit for the service provider, offering incentive for careful management of funds.^6^ In addition, it has been reported that PbR may result in reductions in length of stay and shorter waiting lists for elective procedures.^7^ On the surface, PbR looks beneficial to commissioners, patients and possibly the service providers.

The seeming efficiency in terms of the management of finances, reduction in patient waiting times and shortened length of in-patient stay may not be all it seems. Reservations over taking such consequences at face value are held by the International Council of Nurses,^8^ who fear that such ‘positive’ effects may actually result in patients being discharged too soon (‘quicker and sicker’), placing an extra burden on other support networks. It is possible that the PbR process in acute care does not offer monetary rewards for results as such, but rather it remunerates activity,^1^ and activity that may not be necessary or in the patient's best interest.

### ‘Gaming’ in mental health services

Consistent and significant mismatches between clustering and ICD-10 diagnosis may be down to service providers ‘cherry picking’ cheaper cases and manipulating patient coding into higher tariffs.^9^ These fraudulent processes can be broken down further, illustrating how the system may be manipulated: ‘cream skimming’ or adverse election of lower-cost patients; ‘skimping’ or a reduction in quality of care; ‘up-coding’, which refers to the categorisation of patients into higher-income clusters than what is clinically necessary; and ‘dumping’, which is the selective, inappropriate referring of patients to other care settings.^10^ Such openness to fraudulent processes or ‘gaming’ is of interest to not only commissioners and service providers, but also auditors and researchers.

### Informing funding through diagnosis: problems and international perspectives

The use of diagnosis to inform funding has been questioned in countries that have already rolled out similar schemes.^11^ Mason & Goddard^11^ reviewed the international literature on PbR in mental health along with an economic assessment of the approach in England. It is acknowledged that mental health treatment often extends far beyond the hospital sector and thus, by putting a limit on funding a care package, mental health patients' treatment may be cut short. There is evidence that individuals with mental health problems are more likely to experience physical ill health and are more likely to have greater non-clinical needs, such as educational, social and/or employment support.^12^ The interface between in-patient and community care poses problems in predefining care pathways and currencies, as prognosis and course of treatment is highly variable, regardless of similar mental health diagnoses.

‘Length of stay’ has been found to be a major explanatory variable for cost variation between ‘similar’ patients, which would be particularly relevant for the NHS as a whole, if not for individual service providers, and a fair payment system must be able to compensate by being flexible enough to make appropriate adjustments for patients.^11^ US health providers recognise such variation between mental health patients in both in-patient and outpatient settings and consequently operate a per diem system: there is recognition of the complexities of psychiatric problems coexisting in mental health patients and so funds are distributed on a day-to-day basis, taking into account average costs that are adjusted to account for diagnosis and comorbidity.

Alternatively, the Canadian methodology separates length of stay into three separate parts that are defined through resource intensity, allowing for an adjustment to payments for interrupted stays.^1^ This is a vital consideration as, by comparison, the within-class homogeneity of the Australian and New Zealand mechanisms (the two systems that have the most resonance with the UK's) resulted in the systems never being rolled out to direct funding. However, they were never rolled out to direct funding, in the main because of within-class resource homogeneity. It is extremely difficult to classify resource consumption of different patients even though they may have similar diagnoses.

Both the American and Canadian methodologies account for outliers and facilitate flexibility around length of stay. The conversion of costs to price is not a simple exchange. The English methodology does acknowledge the need for review dates and results in some flexibility. Nevertheless, the date defines cluster episodes and costs, and so the malleability of care clusters may be somewhat limited when compared with its American and Canadian counterparts.

## PbR in forensic mental health

The complexities of care for individuals who have mental health problems make PbR a difficult mechanism to employ with this population. It is noted that even though the rollout of PbR in mental health services has begun, it is still in its infancy. The acute hospital setting had a decade of development and refining. PbR in mental health services has yet to result in subsequent national tariffs and therefore block contracting still informs funding. Such complexities may be further extenuated when a forensic mental health population is considered.

### Development of forensic clusters

The Mental Health Clustering Tool (MHCT)^13^ describes 21 clusters of mental health symptoms and treatment needs observed in general psychiatry. The tool is designed to assess and group individuals according to their clinical needs and resource consumption. It is a pivotal tool in PbR. It has been modified by a group of forensic practitioners to account for risk profiles and personality disorders, in an attempt to make it suitable for a forensic population.^14^ This modified version is called the forensic MHCT.

The modification of the MHCT leaves serious doubts about the suitability of the subsequent forensic MHCT. A multidisciplinary team working in forensic services was convened and split into small groups. They then applied the MHCT to both fictitious and real patients in order to identify actual or possible issues when applying the MHCT to their forensic patients. To the best of our knowledge, there are no statistical underpinnings of the forensic clusters. Only the original clusters, devised through non-forensic samples, have any statistical underpinnings,^15^ and these do not allay concerns over the statistical foundations of the tool.

### Forensic clusters and pathways: research so far

McCann & Green^14^ carried out pilot work to test the utility of the forensic MHCT and another ‘grouping’ instrument developed by forensic practitioners, the Five Forensic Pathways (5FP),^14^ which uses data from the HCR-20,^16^ HoNOS-Secure^17^ and patient's offending history. Small sample size precluded sufficient statistical analysis, making it difficult to draw conclusions from the study.

### Other considerations

#### Adaptability of PbR

It is not yet possible to see whether clustering routes lack specificity with regard to individual needs and resource consumption or whether such routes actually avoid creating complexities that could hinder the applicability of PbR to forensic mental health services. PbR guidelines recognise that patients' needs change over time and that frequent re-assessment and clustering is needed to continually provide individuals with the appropriate care.^18^ The booklet states that ‘lessons are still being learnt’ about how well the PbR system reflects, and how well it accommodates, the dynamic needs of forensic mental health patients, highlighting the need for further research. If it is not known how the system adapts with changing patient needs then it cannot be used to define an individual's care funding.

#### Patient outcomes and effects on funding requirements

Outcome measures are a further consideration in investigating the utility of PbR within forensic mental health. In England, the Department of Health is looking at outcome measures far more now than they did previously.^19^ In non-forensic settings, there is scope for outcome measurements in a set of quality indicators: clinician-related outcome measures (CROMs), patient-reported outcome measures and patient-reported experience measures.^20^ How such outcome measurements transfer to a forensic setting has to be explored. Quality indicators in a forensic setting could include the percentage of patients with a forensic MHCT and 5FP score at admission and subsequent care pathway approach meetings. Similarly, CROMs could include HoNOS-Secure measurements.

However, such proxy measures undertaken when patients are contained cannot reliably inform on how a patient will behave or feel on release into society. Patients discharged from secure care are vulnerable to re-admission, re-offending and mortality,^21,22^ and therefore insight into how clusters and treatment pathways relate to patients' routes after discharge is imperative. A shorter length of stay, for instance, does not portray a positive outcome if the individual in question is subsequently re-admitted or re-convicted as a result of being discharged too soon. A patient's course after discharge should be considered as part of the clustering process, having a role in informing funding and not merely being used as an evaluative tool. Therefore, even though outcome measurements may be transferrable from non-forensic mental health to forensic settings, there are further considerations that should be undertaken due to the nature of the patient population.

## Next steps

The utility of PbR within forensic mental health needs thorough examination. Currently, the only insight has been gained through small preliminary investigations conducted by proponents of PbR. The system has already been rolled out within acute hospital settings and the application of the process to non-forensic mental health is well underway. Even so, the Royal College of Psychiatrists released a statement at the beginning of 2014, expressing concerns over PbR.^23^ More specifically, they highlight reservations over the statistical analyses underpinning the 21 clusters, the range (or lack) of complexity involved in the clustering process, whether the clusters allow for best evidence-based practice, the lack of outcome measures and consequent effect on costs of patient care and ultimately, concerns that the current system would risk severe destabilisation financially and organisationally.

The reservations over the implementation of the mechanism in general mental health generates concern as PbR in forensic mental health is in its comparative infancy. Indeed, the forensic MHCT clusters and the pathways in the 5FP have no statistical underpinnings.

To determine the utility of PbR in forensic mental health, the relationships between diagnoses, care needs assessments and outcomes post-discharge need to be explored. Economic assessments of the treatment costs throughout in-patient and post-discharge accommodation need to be undertaken. There needs to be a profile of economic outcomes for each care cluster if the PbR mechanism is to be rolled out within forensic mental health and ultimately define patient funding. There is a clear and urgent need for research focusing on how the forensic MHCT can be used (if at all) to best cluster patients and what complexities and difficulties exist in the clustering process.
